# Concealment of Preexisting Aortic Regurgitation by the Intimal Flap in Acute Type A Aortic Dissection

**DOI:** 10.7759/cureus.93998

**Published:** 2025-10-07

**Authors:** Shohei Mitta, Yukihiro Matsuno, Yukio Umeda

**Affiliations:** 1 Cardiovascular and Thoracic Surgery, Gifu Prefectural General Medical Center, Gifu, JPN

**Keywords:** aortic dissection, aortic regurgitation, aortic valve prolapse, arch replacement, echocardiography, intimal flap

## Abstract

Aortic regurgitation (AR) frequently accompanies acute type A aortic dissection (ATAAD); however, the underlying mechanism is not always apparent preoperatively. We describe a rare case in which the intimal flap concealed preexisting AR, leading to underestimation of the severity until after surgical repair. A 50-year-old man presented with ATAAD and underwent total arch replacement under moderate hypothermic circulatory arrest. Preoperative and intraoperative transesophageal echocardiography demonstrated only trivial AR. After replacement of the dissected aorta and removal of the intimal flap, severe AR became evident. Long-axis imaging suggested prolapse of the right coronary cusp as the underlying cause. Aortic valve replacement was subsequently performed, and the patient had an uneventful recovery. This case illustrates that the intimal flap can mask the severity of AR in acute dissection, potentially leading to intraoperative diagnostic pitfalls. Careful echocardiographic evaluation, including consideration of valve morphology and jet direction, is crucial for accurate assessment and surgical planning in such patients.

## Introduction

Aortic regurgitation (AR) of varying degrees is reported in up to 70% of patients with acute type A aortic dissection (ATAAD) [1-4]. The mechanisms include dilation of the sinotubular junction, commissural detachment, cusp prolapse, or root involvement caused by dissection [1-4]. In addition, several reports have described rare cases of intimo-intimal intussusception, in which a circumferentially dissected intimal flap prolapses through the aortic valve into the left ventricle, producing significant AR [5-7]. However, to our knowledge, no case has demonstrated an intimal flap concealing preexisting AR, thereby leading to underestimation of its severity until after aortic repair. We report this unusual phenomenon and discuss its diagnostic and surgical implications.

## Case presentation

A 50-year-old man presented with sudden chest pain and left-leg weakness. On arrival, he was hemodynamically stable without altered consciousness. Physical examination revealed motor-sensory disturbance of the left leg and an absent left femoral pulse. Emergency transesophageal echocardiography (TEE) showed concentric left ventricular (LV) hypertrophy, preserved ejection fraction (60%), and trivial AR; a circumferential intimal flap extended into the aortic root. No definite proximal intussusception was seen.

**Figure 1:**
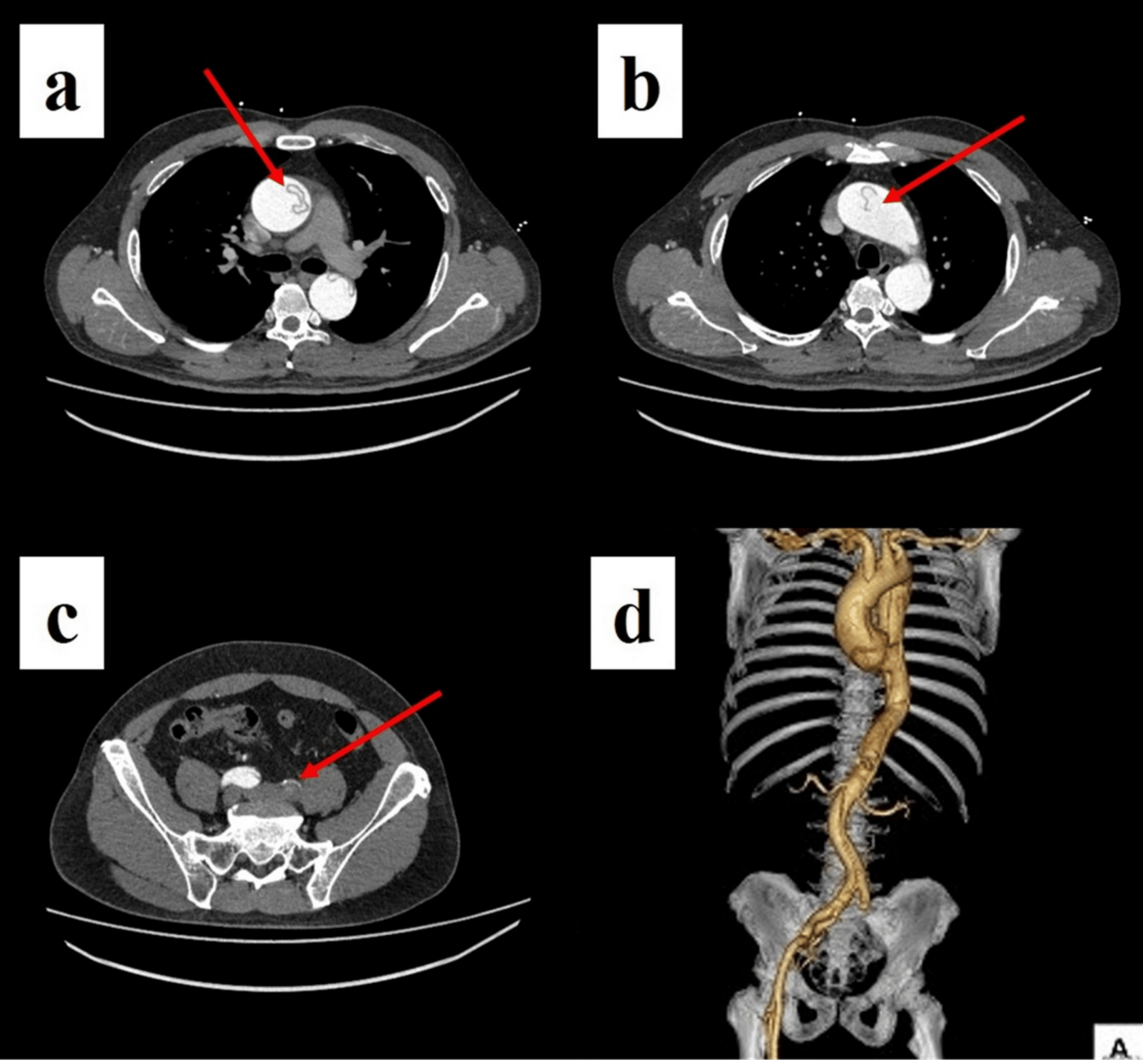
Preoperative contrast-enhanced CT (a, b) Entry tear in the ascending aorta with near-circumferential dissection (arrows). (c) Complete occlusion of the true lumen of the left common iliac artery by a thrombosed false lumen (arrow), consistent with the clinical presentation of left-leg malperfusion. (d) 3D reconstruction clearly demonstrating the longitudinal extent of dissection from the ascending aorta to the femoral arteries.

Contrast-enhanced CT demonstrated dissection from the root to both common iliac arteries. The primary entry was in the distal ascending aorta; the arch branches proximally, and both renal arteries were dissected; the left common iliac true lumen was compressed/occluded by a thrombosed, enlarged false lumen; the celiac and superior mesenteric arteries arose from the true lumen, and the inferior mesenteric from the false lumen (Figure 1).

The diagnosis was ATAAD with left-leg malperfusion.

**Figure 2:**
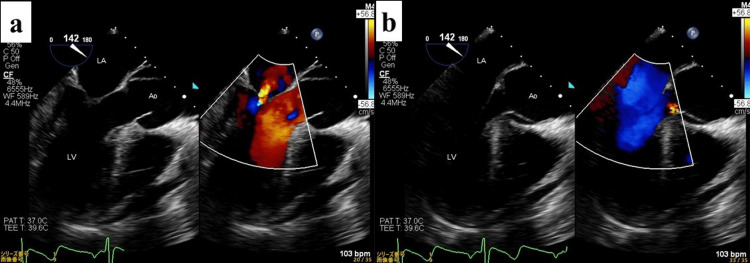
Preoperative TEE (a) Systolic phase. (b) Diastolic phase showing only a faint regurgitant jet into the LV cavity (arrow), consistent with trivial AR at this stage. Ao, aorta; AR, aortic regurgitation; LA, left atrium; LV, left ventricle; TEE, transesophageal echocardiography

In the operating room, pre-procedural TEE by the anesthesiologist again suggested trivial AR (Figure 2).

Bilateral axillary arteries were exposed and side-grafted (8-mm, FUSION Vascular Graft, Getinge, Gothenburg, Sweden) for selective cerebral perfusion (SCP). After median sternotomy, cardiopulmonary bypass (CPB) was established via the right common femoral artery and bilateral axillary grafts, with bicaval venous drainage. A retrograde cardioplegia cannula was inserted into the coronary sinus through a right atriotomy. LV venting via the right superior pulmonary vein followed the onset of hypothermia-induced ventricular fibrillation (VF). Following systemic cooling, at a tympanic temperature of 24°C, the left common carotid artery was reconstructed with an 8-mm graft for antegrade SCP, and circulatory arrest with open distal anastomosis was instituted. Cold blood retrograde cardioplegia was given at 20-minute intervals until coronary perfusion resumed. Pulmonary artery pressures remained unremarkable before and after VF under CPB.

**Figure 3:**
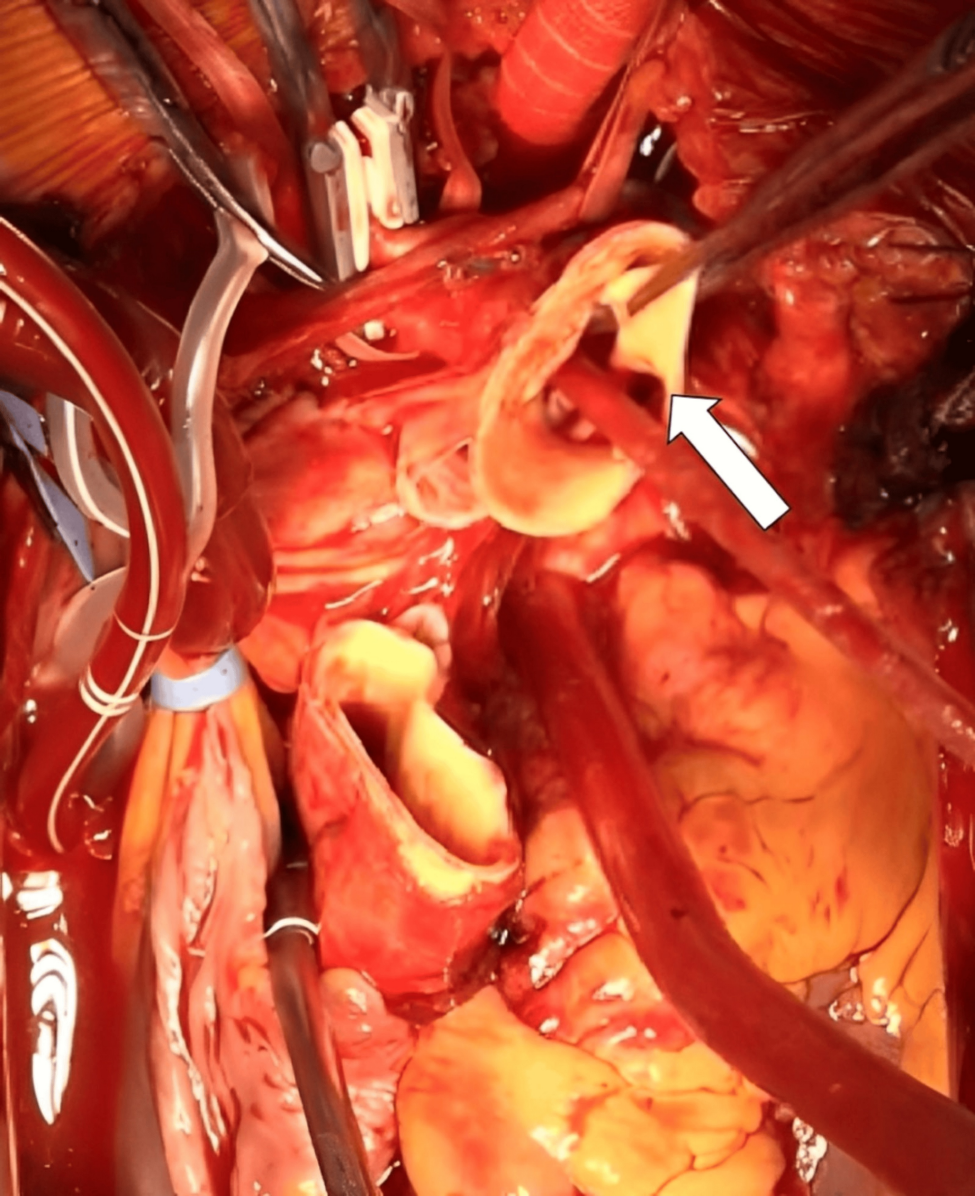
Intraoperative findings Transection of the ascending aorta revealed an almost circumferential dissection with an intimal tear (arrow) presumed to represent the primary entry site near the origin of the brachiocephalic artery.

Operative findings included an almost circumferential intimal tear in the distal ascending aorta near the brachiocephalic origin (Figure 3).

**Figure 4:**
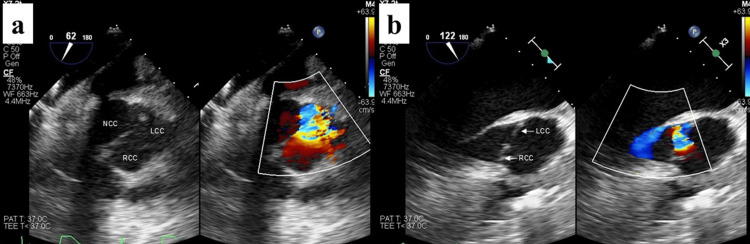
Retrospective review of preoperative 3D TEE (a) Short-axis view demonstrating severe AR. (b) Long-axis view suggesting that the regurgitation is attributable to prolapse of the RCC, as indicated by the direction of the regurgitant jet. Ao, aorta; AR, aortic regurgitation; LA, left atrium; LV, left ventricle; RCC, right coronary cusp; TEE, transesophageal echocardiography

The arch was transected at Zone 1, excluding the tear. An open stent graft (25 mm in diameter, 120 mm in length, FRZX-25120, FROZENIX, Japan Lifeline Co., Ltd., Tokyo, Japan) was deployed, followed by distal anastomosis using a 22 mm four-branched graft (22 mm, J Graft SHIELD NEO^®^, Japan Lifeline Co., Ltd.). Proximally, dissection extended into the non-coronary and right-coronary sinuses; the commissures and coronary ostia appeared intact. The proximal stump was constructed using BioGlue^®^ within the false lumen and an external ePTFE felt strip (15 mm in width, 1.65 mm in thickness, and 150 mm in length) applied with Hydrofit^®^ (Terumo Corporation, Tokyo, Japan). Proximal anastomosis was performed following confirmation of the aortic valve coaptation by water test. However, after graft declamping, TEE revealed prominent AR caused by right coronary cusp (RCC) prolapse, without evidence of recurrent sinus dissection (Figure 4).

**Figure 5:**
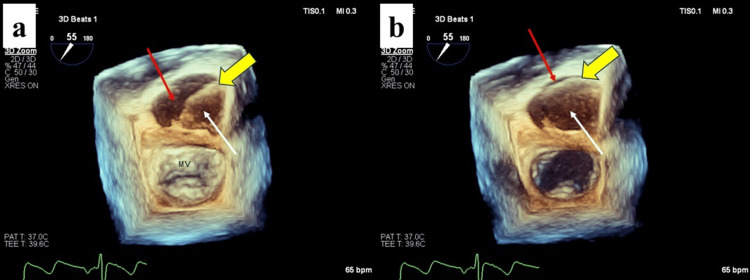
Retrospective review of preoperative 3D TEE (a) Systolic phase. (b) Diastolic phase showing the intimal flap (yellow arrow) covering the aortic valve orifice, acting as a “lid” and concealing severe regurgitation from RCC prolapse. The true lumen is indicated (red arrows), and the false lumen is indicated (white arrows). Ao, aorta; LA, left atrium; LV, left ventricle; RCC, right coronary cusp; TEE, transesophageal echocardiography

Given stable systemic perfusion, arch branch reconstructions (brachiocephalic, left common carotid, and left subclavian arteries via the axillary graft) were completed during rewarming, as no significant elevation in pulmonary artery pressure was observed under LV venting. Upon reevaluation after discontinuing LV venting, severe AR was confirmed, accompanied by LV enlargement and a mean pulmonary artery pressure of 27 mm Hg, necessitating valve intervention. The graft was reclamped, cardioplegic arrest was re-induced, and the proximal anastomosis was taken down. A 22-mm mechanical aortic valve (ATS Open Pivot^®^ Heart Valve, ATS Medical, Inc., Minneapolis, MN, USA) was implanted through the proximal stump. Direct inspection showed no new dissection in the Valsalva sinuses. Retrospective frame-by-frame review of the pre-procedural TEE revealed that the intimal flap fully covered the aortic valve during diastole, acting as a lid and functionally concealing preexisting RCC prolapse (Figure 5).

Left-leg perfusion was satisfactory at the end of surgery; no adjunct limb intervention was required. Postoperatively, he developed myo-nephropathic metabolic syndrome and transient renal failure attributable to preoperative leg ischemia, requiring temporary dialysis. He was extubated on postoperative day (POD) 6, transferred to rehabilitation on POD 31, and remains well with intact repairs at one-year follow-up. Preoperative LV dimensions were not documented. However, postoperative echocardiography revealed LV end-diastolic and end-systolic diameters of 52 mm and 42 mm, respectively, consistent with the hemodynamic burden of severe AR unmasked after surgery. No classical peripheral stigmata of AR (such as wide pulse pressure or Quincke’s sign) were recorded at admission.

## Discussion

AR in ATAAD arises from dissection-induced distortion of the functional annulus, such as STJ/root dilatation, commissural detachment, or leaflet prolapse, and less commonly from proximal flap intussusception through the valve. TEE is pivotal for mechanism-specific surgical planning and for deciding between valve resuspension/preservation versus replacement [1-4].

Most intimo-intimal intussusceptions exacerbate AR by preventing leaflet coaptation or impeding coronary flow [5-11]. In contrast, our case illustrates a diastolic “lid-like” configuration in which an extensively dissected flap completely covered the valve orifice, abbreviating or masking the regurgitant phase and misleading initial TTE/TEE reads under time pressure. While the presence of AR readily draws clinical concern, its apparent absence is often taken at face value, particularly when decisions must be made rapidly. The only echocardiographic finding similar to our case was reported by Chappell et al. [12], in which the aortic insufficiency was limited to the first half of diastole by prolapse of the intimal flap against the regurgitant orifice. However, no case has been described in which AR remained concealed throughout the entire diastolic phase and even during CPB with VF, as observed in our patient. Awareness of this pitfall is crucial when emergency workflows limit the surgeon’s direct review of images.

Contemporary guidance endorses urgent surgery for ATAAD and favors valve resuspension/preservation when leaflet tissue is viable and root geometry can be restored; replacement is appropriate when cusp pathology or root destruction is present [13-16]. In our patient, severe RCC prolapse emerged only after declamping, mandating replacement despite an initially “negative” water test. This highlights that water testing in an unpressurized root can underestimate cusp prolapse that manifests under physiologic diastolic pressure. Where uncertainty persists, pressurized root assessment, using validated intraoperative devices that simulate diastolic loading, can more accurately predict competence both before and after repair [17-19].

Practical recommendations can be drawn from this case. In ATAAD, surgeons should anticipate atypical flap dynamics that may mask the severity of AR. Careful review of key TEE clips by the operating surgeon is essential, particularly diastolic two- and 3D long-axis views obtained across multiple planes. When valve sufficiency is borderline or when imaging tests provide discordant results, a pressurized root evaluation should be performed prior to declamping. Furthermore, the surgical team should be prepared to pivot to valve repair or replacement based on intraoperative reassessment. Devices for root pressurization and direct endoscopic root inspection have demonstrated feasibility and may provide a closer approximation to physiologic evaluation during cardioplegic arrest [17-20].

This case is inherently limited by its design as a single descriptive case report. Quantitative echocardiographic parameters such as regurgitant volume or jet area were not available due to the emergent nature of the presentation. Therefore, while the images and intraoperative findings support our interpretation, the conclusions cannot be generalized. The primary value of this report lies in raising awareness of a rare diagnostic pitfall rather than providing statistical inference.

## Conclusions

We encountered a rare case in which severe AR due to RCC prolapse was functionally concealed by an intimal flap in ATAAD. Although AR is commonly highlighted as a critical determinant of surgical urgency in ATAAD, this case illustrates that its apparent “absence” may be misleading in the emergent setting. In particular, diastolic coverage of the valve orifice by a circumferentially dissected flap can act as a lid, masking preexisting AR and leading to intraoperative underestimation.

As a single case report, our findings should be interpreted with caution and cannot be generalized statistically. However, the educational value is significant: greater awareness of this “concealment phenomenon” may prevent delays in valve intervention, improve surgical planning, and ultimately enhance outcomes in this high-risk population.
